# Topical Treatment for Retinal Degenerative Pathologies: A Systematic Review

**DOI:** 10.3390/ijms24098045

**Published:** 2023-04-28

**Authors:** Lăcrămioara Samoilă, Oliviu Voștinaru, Elena Dinte, Andreea Elena Bodoki, Bogdan-Cezar Iacob, Ede Bodoki, Ovidiu Samoilă

**Affiliations:** 1 Department of Physiology, “Iuliu Hatieganu” University of Medicine & Pharmacy, 400006 Cluj-Napoca, Romania; 2 Department of Pharmacology, Physiology and Physiopathology, “Iuliu Hatieganu” University of Medicine & Pharmacy, 400349 Cluj-Napoca, Romania; 3 Department of Pharmaceutical Technology and Biopharmaceutics, “Iuliu Hatieganu” University of Medicine & Pharmacy, 400012 Cluj-Napoca, Romania; 4 Department of General and Inorganic Chemistry, “Iuliu Hatieganu” University of Medicine and Pharmacy, 400010 Cluj-Napoca, Romania; 5 Department of Analytical Chemistry, “Iuliu Hatieganu” University of Medicine & Pharmacy, 400349 Cluj-Napoca, Romania; 6 Department of Ophthalmology, “Iuliu Hatieganu” University of Medicine & Pharmacy, 400006 Cluj-Napoca, Romania

**Keywords:** topical treatment, eyedrops, retina, clinical studies, age related macular degeneration, macular edema

## Abstract

The topical administration of medicines is the preferred route in ocular therapy, at least for the anterior segment of the eye. However, the eye’s inherent functional and biological barriers all work against the active pharmaceutical ingredient (API) to efficiently reach the targeted retinal structures. The main objective of this article is to offer a systematic review of the scientific literature in recent years, focusing on the latest developments of topical treatment intended for retinal degenerative diseases. Database search returned 102 clinical studies, focused on topical treatment for age macular degeneration, macular edemas (in diabetic retinopathy, surgery related or in retinal dystrophies) or glaucoma. After the exclusion of low-powered studies and those combining vitreo-retinal surgery, 35 articles remained for analysis. Currently, the topical treatment of retinal degenerative diseases is limited by the difficulty to deliver effective drug concentrations to the posterior eye structures. However, in the case of drug classes like NSAIDs, the presence of certain molecular and metabolic features for specific representatives makes the topical administration currently feasible in several clinical contexts. For other drug classes, either a fine-tuning of the API’s pharmacokinetic profile or the use of more advanced formulation strategies, such as rationally designed nanostructured drugs and vehicles, crystalline polymorphs or supramolecular complexes, could bring the much awaited breakthrough for a more predictable and controlled delivery towards the retinal structures and could eventually be employed in the future for the development of more effective ways of delivering drugs to the posterior eye, with the ultimate goal of improving their clinical efficacy.

## 1. Introduction

The complexity of the eye is probably unmatched in the human body, even by the brain itself. Different mechanisms operate together for the formation of images. Optical, structural, muscular and neural components work simultaneously to keep the object in focus and to generate electric patterns interpreted by the brain into images. It is a well-calibrated system, which uses large amounts of energy, and it is stimulated by large amounts of energy, in the form of light.

The immune privilege of the eye has the purpose of keeping all these components intact and the optical axis transparent. The functional integrity of the eye is maintained by a series of barriers, both external and internal. There is also a particular immunological response of the eye.

The topical application of treatment onto the eye surface is the preferred route in ocular drug administration, by clinicians and patients alike [1,2]. However, these biological and functional barriers of the eye prevent most of the drug from reaching its target. Less than 5% of the active pharmaceutical ingredient (API) applied topically reaches the inner structures of the eye [3,4]. External barriers of the eye are found on the eye’s surface, while the internal barriers are present at the retina.

### 1.1. Eye Barriers

#### 1.1.1. External Barriers

When using eye drops, the first barrier is the blinking system. The drops (or any other pharmaceutical forms, e.g., gels, ointments) are placed into the conjunctival sac, beneath the lower eyelid. The volume of the eye drops varies from 25 to 70 microliters, but the tear film has a volume of 7 microliters, and the ocular surface can only retain a maximum of 30 microliters of liquid [5], without blinking. The blink after the instillation will wash out most of the instilled eye drop. The lacrimal system will wash some of the volume into the lacrimal drainage system, through lacrimal punctum into lacrimal canaliculi, lacrimal sac and eventually into the nose. Lacrimation reflex after instillation may also be induced, increasing the lacrimal clearance.

The small amounts of drug still remaining on the eye’s surface will have the difficult task of penetrating the corneal epithelium. The cornea consists of five different layers. Among them, the epithelium (especially) and the stroma are the most important corneal barriers. The epithelium consists of cells linked together into tight junctions, not easily penetrated by large molecules. Epithelium is hydrophobic, while the stroma is hydrophilic. Lipophilic agents, in principle, penetrate better through the cornea. Inside the eye, in the anterior chamber, there is an aqueous outflow, which will flush the xenobiotic towards the irido-corneal angle, into the aqueous drainage system, and outside the eye. The crystallin lens follows in the series of biological barriers in front of the vitreous body. In a pure axial manner of ocular biointernalization, the APIs will have to pass through cornea, aqueous humor, crystallin and vitreous body to reach the retina.

Another trajectory for the ocular internalization of the topically administered bioactives formulated as eye drops is transconjunctival, followed by the transscleral route. Hydrophilic agents have a better penetration through this route. The conjunctiva is more permeable than the cornea [6]. However, the conjunctiva is highly vascularized, and the drug molecules reaching it are easily transferred into the systemic circulation. The scleral stroma is thicker, paucivascular and functions as a barrier, but the large surface area of the sclera could be important for drug delivery to the posterior segment of the eye. Parabulbar, subtenon or subconjunctival injections, circumventing some of the aforementioned barriers, will deliver the APIs into the inner structures of the eye also through the transscleral route. Inside the eye, the drug may reach the highly vascularized choroid and, subsequently, the external retina or will penetrate the vitreous body on its route towards the inner retina.

#### 1.1.2. Internal Barriers

The retina is a part of the central nervous system, benefiting from the same protection as the brain (through the blood-retinal barrier, or the internal retinal barrier). The vascularization of the retina is provided through the central retinal artery and vein, which, however, provide nutrients only for the inner part of the retina. The inner surface of the retina is in contact with the vitreous body, an avascular structure. An internal limiting membrane will provide a barrier (however weak) between the retina and the vitreous body. The outer retinal surface, where the photoreceptors are located, is separated from the rich vascularized choroid by the retinal pigment epithelium (the external retinal barrier). The vascular route of any drug will provide little bioavailability of the substance inside the retina. In the transconjunctival route, drugs will also have to pass through this external retinal barrier.

### 1.2. Drug Delivery for Retinal Pathology

The retina is placed in the focus of the lenses of the eye. The high levels of light, together with the very processes of vision, in high demand of energy and oxygen, place the retina under various stresses—oxidative stress, inflammation. The prevalence of retinal diseases related to oxidative damage is very high. Age-related macular degeneration (AMD), with its two forms, wet and dry, was found in 2019 in 18 million people, in the United States alone [7]. Among them, 1.5 million had advanced form AMD and very low vision. The number of people living with AMD is estimated to reach 288 million worldwide by 2040 [8]. Diabetic retinopathy is found in one-half of the diabetic patients, after 10 years of evolution of the disease, especially in cases of metabolic imbalance (glycated hemoglobin higher than 7%). Glaucoma is estimated to impact 57.5 million people worldwide [9]. AMD, glaucoma and diabetic retinopathy are found among the main causes of vision loss in the developed world. Retinal inherited dystrophies, i.e., retinitis pigmentosa, Best and Stargardt disease also share oxidative damage mechanisms. Lately, age related neurological disorders, like Alzheimer’s disease, have been found to induce retinal alterations from early-stage (Aβ deposition, neurofibrillary tangle aggregation, abnormal retinal microvascular circulation and thinning of the retinal nerve fiber layer) [10].

All these retinal diseases are considered to benefit from antioxidant supplementation, which is currently included in the standard treatment. The route of administration is so far systemic. Lutein, Zeaxanthin (and metabolites: Mesozeaxanthin and Astaxanthin) and vitamins C and E are usually found together in capsules formulated from AREDS studies, for oral administration [11].

In different stages of AMD and diabetic retinopathy, or in the case of any other retinal disease progressing with macular edema, Anti-VEGF treatment is also indicated although a more invasive route of administration, through intravitreal (IVT) injections, is commonly approached.

Finally, anti-inflammatory drugs can be prescribed in certain pathologies of the macula (central serous chorioretinopathy or diabetic macular edema—DME). Moreover, cystoid macular edema (CME) is another important cause of vision loss after ocular surgical procedures. In fact, the results of a study showed that CME has been reported in 19% of patients subjected to modern, small-incision cataract surgery, using fluorescein angiography criteria [12].

Table 1 highlights the routes of administration for different medicines in today’s practice, in degenerative retinal pathology, and the barriers for drug permeation to target. The main disadvantages are also highlighted.

Anti-inflammatory drugs are administered topically (Bromfenac, Nepafenac), via intravitreal (Triamcinolon), parabular or subtenon injection (corticosteroids) and systemically (oral NSAIDs, oral or i.v. corticosteroids). Low bioavailability is the main limitation, especially in topical administration, while systemic administration frequently causes systemic adverse reactions.

Intravitreal injections seem to provide the best retinal access for all drug molecules, in general, although this procedure is invasive and associated with certain risks (intraocular infection, retinal detachment, etc.). Usually, very small amounts of the drug are necessary in the injection (0.05 to 0.1 mL, consisting of less than 1 mg active substance, in the case of Anti-VEGF drugs).

**Figure 1:**
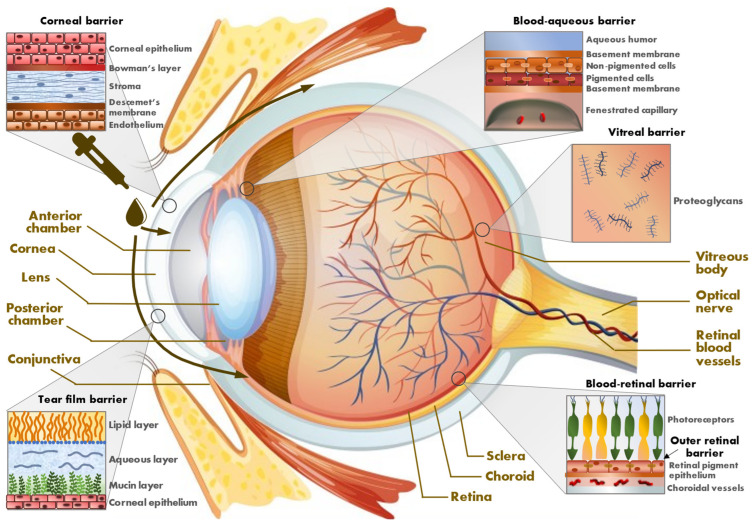
Biological barriers shaping the route of APIs upon topical treatment of the eye.

The biological barriers upon topical treatment encountered by the APIs following either the corneal or the transconjunctival route are depicted in Figure 1.

This review focuses on the topical treatment targeting different retinal diseases having in common oxidative stress and inflammation, from a clinical point of view, including only medicines that reached into clinical trials. Previous reviews documented various routes for drug delivery, with only tangential references to topical route [13]; others presented only new technologies (e.g., nanotherapies [14] or Molecularly Imprinted Polymer-Based Drug Delivery Systems [15]); and others investigated only selective ocular diseases (e.g., AMD and glaucoma [2]).

## 2. Methods

**Figure 2:**
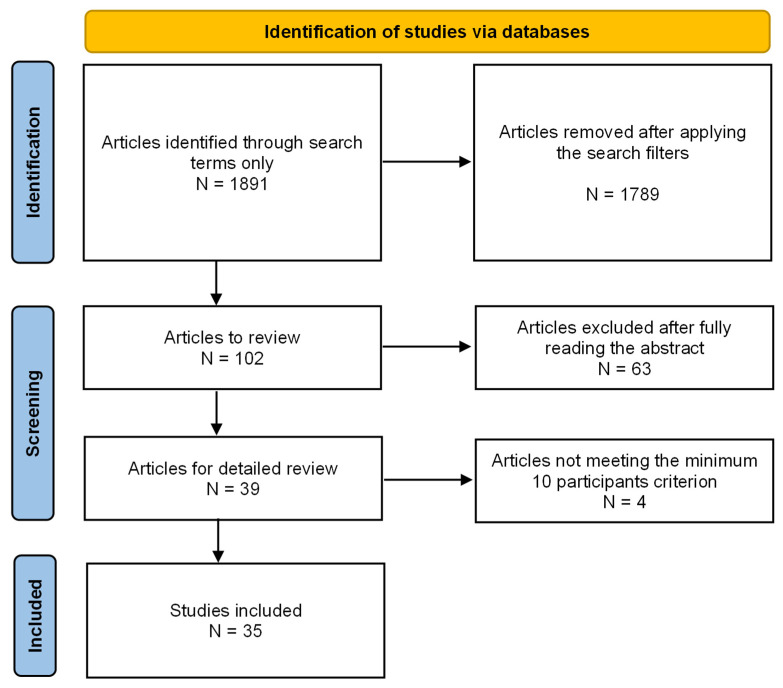
Flowchart of the reported studies’ selection.

The main objective of this article is to provide a systematic review of the scientific literature in recent years, focusing on the recent developments of topical treatment intended for retinal degenerative diseases. Database search included PubMed/Medline (National Library of Medicine, Bethesda, MD, USA) and Scopus (Elsevier, Netherlands), starting 2010 until 2023, with mesh terms “Topical and Retina”, in English language. The search returned 1891 results and 102 clinical studies (Figure 2).

Inclusion criteria were established: focus only on degenerative retinal disease or oxidative stress-related damage, at least 10 patients enrolled in the study, no previous posterior vitrectomy surgery. Eyes with macular edema (CME) after cataract surgical procedures were also included, considering the presence of mechanisms common to degenerative pathologies (oxidative stress, inflammation). Posterior vitrectomy removes the vitreous body, changing the pharmacodynamics of any drug reaching the eye. After applying the aforementioned criteria, 35 studies were finally included. One case of Alzheimer’s disease was also included [16] as it investigated changes related to oxidative stress at the level of the retina.

The outcome of topical treatment was usually measured in a comparable fashion. Most studies reported change in best corrected visual acuity (BCVA) and ocular coherence tomography (OCT)—especially changes in central retinal thickness (CRT). Some studies focused on functional gains (microperimetry) or electrophysiological changes (visual evoked potentials—VEP; electroretinogram—ERG). In total, 2262 eyes were investigated.

## 3. Results

**Figure 3:**
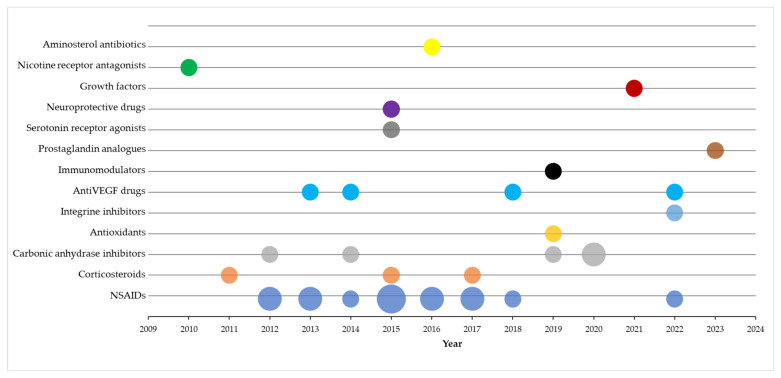
Timeline for clinical studies investigating topical treatment in retinal degenerative diseases. The circle size is proportional to the number of publications per year.

The clinical trials included in the review investigated 13 classes of medicines in topical administration: NSAIDs (Bromfenac, Nepafenac, Ketorolac, Diclofenac); Corticosteroids (Prednisolone, Dexamethasone); Carbonic anhydrase inhibitors (Dorzolamide); Antioxidants (Coenzyme Q10); Integrine inhibitors (OTT166); Anti-VEGF drugs (Regorafenib, Acrizanib, Pazopanib); Immunomodulators (Interferon); Prostaglandin analogues (Unoprostone); Serotonin receptor agonists (Tandospirone); Neuroprotective drugs (Citicoline); Growth factors (Human nerve growth factor); Nicotine receptor antagonists (Mecamylamine); and Aminosterol antibiotics (Squalamine). The majority of clinical trials focused on topical use of NSAIDs (15 studies), carbonic anhydrase inhibitors (5 studies) and Anti-VEGF (4 studies). The timeline for these studies is illustrated in Figure 3.

The topical treatment targeted diseases located in the macula (dry and wet AMD; CME from different causes—in retinitis pigmentosa, after central retinal vein occlusion and after cataract surgery; DME; Chronic Central Serous Chorioretinopathy), diseases inducing retinal photoreceptor changes (diabetic retinopathy, retinitis pigmentosa, Alzheimer’s), diseases changing the retinal vessels (diabetic retinopathy) and diseases of the optic nerve (glaucoma).

Table 2 represents a synthesis of the 35 clinical trials included in the review, in chronological order, grouped in classes of APIs. Investigated drug and regimen, proposed mechanism, studied pathologies and declared clinical success were also noted. Clinical success was considered when all the main outcomes of the study were met (e.g., BCVA, OCT-CRT). Partial success was noted when one of the main outcomes was not met (usually BCVA), and finally no success was noted when none of the main outcomes were met.

## 4. Discussion

The clinical effectiveness of an ophthalmic drug is influenced by three main factors: (i) the nature and number of physiological barriers of the eye to be overcome by the API; (ii) the particularities of the pharmacodynamic and pharmacokinetic profile of the API (related to properties of the drug such as lipid solubility, polarity and ionization degree); and (iii) the characteristics of the pharmaceutical dosage form in which the active molecule is incorporated.

Considering the difficulty of obtaining effective concentrations at the posterior segment of the eye, intravitreal injections would seem the optimal choice to achieve the much-desired therapeutical goal at this level. This route of administration is not, however, void of risk, and in case of long-term treatments, it requires careful timing between consecutive injections or administration of prodrugs, reservoir-type or controlled-release pharmaceutical systems (nanoformulations, implants) able to ensure therapeutical levels of API for prolonged periods of time. The formulation of these complex systems involves the use of excipients that lack toxicity, which significantly limits the choice of these auxiliary ingredients; that in turn implies constraints in the development of these dosage forms.

Clinical studies suggest that the IVT administration is advantageous in the case of molecules with high molecular weight demonstrating poor ocular bioavailability upon topical administration. Current trends, however, aim for the development of topical pharmaceutical forms able to ensure therapeutically effective concentration at retinal level, for a variety of classes of APIs.

Despite all the limitations of the topical route of administration, studies included in the current review have demonstrated the absorption of some APIs up to the level of the retina, the routes involved being corneal or non-corneal (transconjunctival and transscleral). The corneal pathway ensures the absorption of small lipophilic molecules, which reach effective concentrations in the aqueous humor, while the exposure of drugs on the conjunctiva ensured lower concentrations in the aqueous humor but allowed the delivery of molecules to the posterior eye, this being observed even in the case of some larger molecules [42]. Studies on the localization of APIs on the conjunctiva, through the use of technologies that ensure a prolonged residence time at this level, such as contact lenses, have demonstrated the sustained release of ofloxacin, obtaining concentrations of 10–40 times higher compared to the application on cornea [51,52].

Although the concentrations of APIs in the vitreous after topical administration are 10–100 times lower compared to the aqueous humor and the cornea, the use of sensitive analytical methods enable their accurate quantification [53].

The concentration of some molecules in the posterior eye can also be explained by their particularity of preferentially targeting the choroid or the retina, the mechanism that best explains this behavior being the binding to melanin [54]. As an example, brimonidine binds strongly to the pigmented tissues around the vitreous and ensures the activation of neuroprotective receptors from retina. After one week of administration as ophthalmic drops, brimonidine was detected in the vitreous in a concentration of over 2 nM, reaching the range of clinically effective concentrations [55].

Surprisingly, even if ophthalmic drops are considered a dosage form that does not ensure clinical efficiency in the treatment of posterior eye conditions, experimental studies on animal models have demonstrated the presence of APIs in the vitreous or retina [56], while the possibility of ocular distribution also in humans is being supported by rabbit and monkey studies [54,57,58,59].

### 4.1. Anti-Inflammatory Drugs

Topical application is a convenient method to deliver medicines to the eye, as it ensures the highest patient compliance and also enables self-administration. However, the bioavailability of the medicine is very low, and the effectiveness in attaining therapeutic API levels is usually limited to the surface of the eye. Nevertheless, there are some exceptions. Reports indicate some NSAIDs to penetrate better inside the eye, especially Nepafenac. The corneal absorption of a drug depends on its lipid solubility and inversely on its polarity or degree of ionization [60]. To effectively target retinal tissue and to subsequently be able to prevent macular oedema, NSAIDs must reach significant concentrations in the posterior chamber of the eye. Nepafenac has a unique pro-drug structure which favors a superior corneal permeability, compared to other NSAIDs [61]. After topical administration to the eye, nepafenac is transformed into amfenac by intraocular hydrolases, especially in the iris, ciliary body and retina, making it a “targeted” NSAID [62].

On the other hand, Diclofenac, Ketorolac and Bromfenac are relatively water-soluble phenylalkanoic (Ketorolac) and phenylacetic (Diclofenac, Bromfenac) acids and should have limited ability to penetrate corneal epithelium. These are weakly acidic molecules, which ionize at the tear’s pH and thus penetrate the cornea with difficulty. The formulation of topical ophthalmic dosage forms with NSAIDs is limited to the use of compounds with higher solubility in water (indole acetic, aryl acetic and aryl propionic acid derivatives) while adjusting the pH of the formulation to an acidic value that favors the fraction of the non-ionized form, eventually leading to the increase of their absorption rate. Nevertheless, the acidic nature of the NSAID molecules and the acidic pH of the formulation could be irritating to the eye, through chronic use [20].

NSAIDs represent a heterogeneous group of compounds with different physicochemical characteristics, being approved by the FDA for use in various eye diseases to be administered by various routes. The clinical efficiency and absorption in the posterior segment are still controversial, with some studies demonstrating increased concentrations in the vitreous after topical application, results that can be supported by the possibility of absorption through the mucosal surfaces [63]. In addition, the absorption of molecules through membranes is conditioned by the particularity of the molecule. Thus, the more obvious clinical efficiency and increased concentration of nepafenac (six times higher compared to diclofenac), a noncharged molecule, is due to the increased permeability through membranes, followed by the formation of the more active form, amfenac, under the action of ocular hydrolases [20].

Bromfenac, a molecule with a similar chemical structure to amfenac (exception of a bromine atom at the C4 position) showed increased penetrability, a fact that prolongs its cyclooxygenase-2 (COX2) inhibitory action [64,65]. Bromfenac exerts the greatest action on cyclooxygenase 1 (COX1), while amfenac is the most potent on COX2. In an experimental retinochoroidal inflammation in rabbits, topical Bromfenac was showed to penetrate into retinochoroidal tissue in concentrations high enough to target COX2 and inhibit its effect on blood-retinal barrier [66].

In vitro studies showed that Nepafenac pro-drug readily penetrates the rabbit cornea well and distributes in all ocular tissues, including aqueous humor, iris, ciliary body, retina and choroid [67]. Predicted levels of NSAID corneal penetration are not precisely in line with those found in medical practice. Bucci et al. [68], in 2007, found that aqueous levels of Ketorolac, following topical ophthalmic administration in humans, far exceed that of amfenac or nepafenac following 2 days of 4-times-a-day drug administration.

In specific clinical situations like cataract surgical trauma, local cyclooxygenases could be excessively activated, leading to high levels of prostaglandins within the eye’s structures. These pro-inflammatory molecules can cause pain and discomfort to the eye, their inhibition with non-steroidal anti-inflammatory drugs being common after ocular surgery. Moreover, anti-inflammatory drugs proved also a favorable effect on visual outcomes [69].

**Figure 4:**
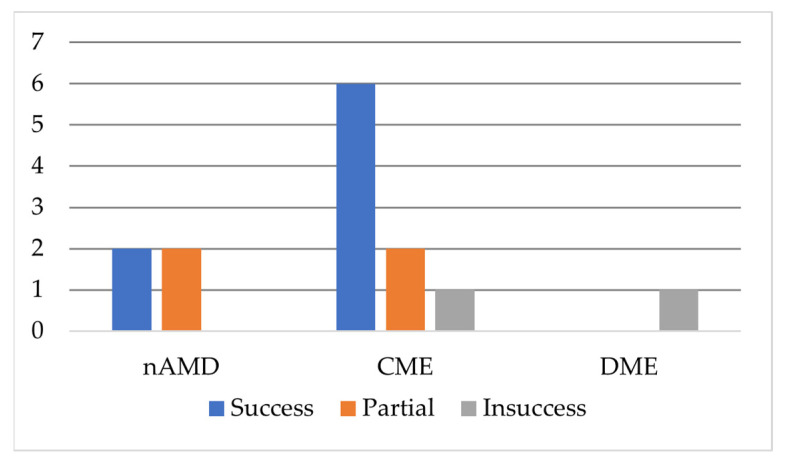
Clinical outcome of NSAID topical treatment of retinal diseases.

Among the 14 NSAID included studies, 8 reported clinical success (Figure 4). This included lower incidence of CME, better BCVA and thinner central retinas (on OCT-CRT). Four studies reported partial success (usually similar BCVA between studied group and control, similar OCT-CRT, no change in the need for IVT injections). NSAIDs were investigated for 3 potential uses on posterior pole, nAMD (4 studies), DME (1 study) and CME related to cataract surgery (9 studies). The success in decreasing macular thickness in nAMD (Figure 3) suggests that NSAID mechanism is not limited to the anterior pole of the eye. In the chase of preventing surgical induced CME, the effect of NSAID on prostaglandin synthesis at the anterior pole, at the iris, may prove to be sufficient to lower prostaglandin levels reaching the macula. The (at least partial) success treating nAMD may prove that NSAID can reach the macula itself. A transconjunctival, transscleral route may be implicated in the effect. Gomi [18] found that NSAID supplementation to Anti-VEGF significantly decreased the need for intravitreal injections. Flaxel [17], Russo [20] and Wyględowska-Promieńska [21] found better OCT-CRT after NSAID supplementation, though no change for the need of IVT Anti-VEGF. The latter also found better BCVA.

Kessel et al. [70] performed a systematic review in 2014 to compare the efficacy of NSAIDs (diclofenac, nepafenac, ketorolac and bromfenac) versus corticosteroids (dexamethasone, betamethasone and fluorometholone). They concluded that topical NSAIDs are more effective than corticosteroids in preventing inflammation (low to moderate quality) and reducing the prevalence of CME after uncomplicated phacoemulsification (high-quality).

Topical administration of Corticosteroids is the preferred route targeting the anterior segment of the eye, the drug reaching the anterior chamber 15–30 min after administration [71]. The main barrier remains the lipophilic corneal epithelium, especially for their more polar and hydrophilic derivatives, such as their phosphate esters (i.e., prednisolone phosphate), but less for their unesterified or acetate esters (i.e., dexamethasone or prednisolone acetate) [72].

Corticosteroids provide powerful anti-inflammatory and anti-edematous effects by targeting not only the synthesis of proinflammatory mediators involved in DME (IL-6, IL-8, MCP-1, ICAM-1, TNF-α, HGF, ANGPT2, etc.) but also a decrease in VEGF synthesis. The only clinical trial included in the review found no benefit in high dose Prednisolone for preventing CME after surgery. BCVA, retinal thickness on OCT-CRT remained similar to low dose corticosteroid regimen. This may point to low penetrability of Prednisolone alone through the cornea.

Two clinical studies incorporating corticosteroids in microparticles were also included in the review. With their lipophilic central cavity and their hydrophilic outer surface, cyclodextrins and their derivatives can be successfully used for the preparation of aqueous eye drop containing corticosteroids (i.e., Dexamethasone). Their supramolecular complexes with cyclodextrins were proven to increase water solubility and stability, and the drug permeability through biological membranes and hence allowed for an efficient delivery of the drug to the retina and vitreous humor [73,74,75,76].

It was proposed that drug-cyclodextrin complexes can aggregate in aqueous media to form nano- or microparticles, the latter being broken into nanoscale structures and even single drug-cyclodextrin complexes once they reach the surface of the eye [77,78]. Furthermore, a mucoadhesive effect that delays dexamethasone’s clearance may contribute to the high and extended remanence of the API in the tear film [77,79]. Also, it was hypothesized that the nanoparticles may even act as delivery vectors that can penetrate mucus and translocate Dexamethasone from the surface of the lipophilic membrane barrier [77].

In an animal model (rabbits), microparticles of dexamethasone-ɣ-cyclodextrin with increased residence time on the eye surface were evaluated for the delivery of the corticosteroid, and the results showed that Dexamethasone’s concentration in the posterior segment of the eye (vitreous and retina) was comparable with the concentrations achieved from the intravitreal injection of the corticosteroid [75]. In a clinical setup, Tanito et al. [32] and a few years later Ohira et al. [33] investigated the effect of topical micro/nano-particles ɣ-cyclodextrin dexamethasone eye drops for DME. Although these studies included a very low number of eyes (19 and 12, respectively), both reported success in DME management, with significant effects on the posterior segment of the eye translated in BCVA increase and OCT-CRT decrease. Furthermore, the clinical results reported by Ohira were comparable to those obtained by subtenon injection of Triamcinolone.

Related to corticosteroid topical use, but not included in this review, Niffenegger et al. [80] observed hole closure in 89% of secondary macular holes with CME after Difluprednate 0.05% treatment for 6 weeks. However, they added topical carbonic anhydrase inhibitor (dorzolamide 2% or brinzolamide 1%) in 6 eyes, and Bromfenac in 2 eyes. (Only 9 eyes were included in this study and the article did not meet the inclusion criteria for present review.) The treated group was too small to draw better conclusion regarding the efficacy of the topical treatment for macular holes.

Another study that did not meet the inclusion criteria investigated Nepafenac for the prevention of CME after pars plana vitrectomy. Mandelcorn et al. [81] compared Nepafenac to Triamcinolone IVT and control and found no success, with similar OCT-CRT in all 3 groups. Vitrectomy eliminates the vitreous body and with that also the proinflammatory cytokines. This changes the inflammatory pathway, being a main reason for considering vitrectomy in the exclusion criteria of the review. An argument to this matter is also the lack of efficacy of Triamcinolone IVT, injected directly to the retina of vitrectomized eyes. Furthermore, the efflux of any substance injected in the vitreous cavity after vitrectomy is increased. For instance, intravitreal Triamcinolone decreases more rapidly in vitrectomized eyes [82], at a rate 1.5 faster than in nonvitrectomized eyes. The half-life of Triamcinolone acetonide was 1.57 days in the vitrectomized group and 2.89 days in the nonvitrectomized group. Regarding Anti-VEGF injections, most animal models indicate that intravitreal drugs have reduced half-lives and increased clearance in vitrectomized eyes [83].

Naithani et al. [84] also investigated Nepafenac after posterior vitrectomy. Topical Nepafenac was safe and reduced postoperative pain and inflammation in patients undergoing vitreoretinal surgery. However, its effect on reducing postoperative macular edema and improving visual acuity as compared with that of the standard post vitrectomy therapeutic regimen was equivocal.

### 4.2. Carbonic Anhydrase Inhibitors

Topical Carbonic Anhydrase inhibitor (Dorzolamide) was investigated in 5 clinical studies (retinal vessels effect in normal versus diabetic population; CME in retinitis pigmentosa; nAMD). All reported success (total—OCT-CRT and BCVA increase, or partial—BCVA increase). Tilma et al. [36] found that Dorzolamide had a vasodilatation effect on normal retinas, while diabetic retinas showed no response. The authors suggest that the dilated state of retinal vessels in diabetic patients is already reached, and medical supplementation would have no further effect. The effect on CME (Reis et al. [35], Ikeda et al. [34]) shows that Dorzolamide does reach the retina in therapeutic amounts.

Dorzolamide in slightly acidic solution is usually used for glaucoma treatment [85]. It is sufficiently hydrophilic to be soluble in the tear film and sufficiently lipophilic to permeate the cornea. The main target is the Carbonic Anhydrase at the ciliary body. At the retinal target, Dorzolamide improves the ability of the RPE to pump fluid out of the retina by modulation of the polarized distribution of membrane-bound Carbonic Anhydrase, at the level of the RPE.

Animal studies suggested that Dorzolamide penetrates to the retina, with high concentrations of the drug 1 and 2 h after topical administration [86]. Kadam et al. [87] investigated Dorzolamide distribution inside the eye. They found that the drug has higher distribution at the anterior part of the eye (aqueous humor, anterior vitreous) but with good levels at the posterior part (posterior vitreous, optic nerve, sclera, choroid, RPE). A mainly transconjunctival route to reach the retina is probable, but both corneal and conjunctival routes are used [88].

Several theories exist as to how Dorzolamide-timolol may be helping in nAMD. The effect on carbonic anhydrase together with β-blockade from Timolol is a potent decrease of aqueous humor flow, hence turnover of intraocular fluid may be slower, delaying drug clearance. Mouse models also suggested the role of the β-adrenergic pathway in VEGF upregulation [38].

### 4.3. Anti-VEGF

Anti-VEGF IVT injections are the main treatment regimen in nAMD. Repeated dosage is usually needed (1×/months). The shift to topical administration is a demand from both ophthalmic specialists and patients. Four studies were included in the present review, investigating Pazopanib, Regorafenib and Acrizanib in topical formulations. Danis et al. [40] found Pazopanib efficacy in genetic subtypes of nAMD. Both BCVA and OCT-CRT improved in patients with Complement Factor H, T allele. The results were not duplicated later [41]. In the case of Regorafenib, in the Joussen et al. [42] trial, the program was terminated after phase IIa because efficacy was lower than it was with current nAMD treatments. According to elaborate post hoc analyses, the most likely reason was the insufficient exposure in the target compartment (back of the eye). In fact, the lack of efficiency of topically applied anti-VEGF drugs, such as Regorafenib and Pazopanib in humans, despite promising preclinical results in rats, was further investigated by Horita et al. [89], who found that the two drugs failed to generate significant concentrations in posterior eye structures in rabbits and monkeys. The study also showed that the concentrations of Regorafenib and Pazopanib in the anterior eye tissues were clearly superior to choroidal and retinal concentrations; therefore, new methods of improving ocular bioavailability of the tested anti-VEGF drugs were investigated. Thus, a nano-crystalized formulation could more efficiently penetrate the eye structures with higher concentrations towards the retina, but further research is needed to clarify this issue [89]. In conclusion, no definite Anti-VEGF topical regimen is advised today.

### 4.4. Integrin Inhibitors

Integrin inhibitor (OTT166) decreases angiogenesis, exudation, inflammation and fibrosis (binding integrins including αvβ3, αvβ6 and αvβ8). Due to its physiochemical properties, including incorporation of fluorine atoms in specific positions on the molecule, OTT166 penetrates the conjunctiva and traverses the sclera and choroid, distributing to the retina upon topical application. OTT166 has demonstrated pharmacologic effects in animal models of neovascularization, vascular leakage and angiogenesis. Boyer [39] found a subgroup of patients (37% from total), responders to OTT166, describing improvements in OCT-CRT.

### 4.5. Citicoline

Citicoline, a neuroenhancer (stabilizes cell membranes by increasing phosphatidylcholine and sphingomyelin synthesis) is also currently available for topical treatment. It is documented that, in an animal experimental model, Citicoline reaches the vitreous (using as vehicles high molecular weight hyaluronic acid and benzalkonium chloride as penetration enhancers) [41]; therefore, there is a real possibility that this substance may act directly on those ocular elements close to the vitreous chamber (ganglion cells and their fibers) that are morpho-functionally affected in glaucomatous disease. In the study of Parisi [46], Citicoline improved VEP and ERG (both amplitude and latency) in glaucoma patients, with values returning to normal 2 months after washout.

### 4.6. Tandospirone

Another neuroprotector, Tandospirone, had good results on animal models. In albino and pigmented rats subjected to a severe acute photo-oxidative stress, Tandospirone protected photoreceptors and RPE cells in a dose dependent manner. In Jaffe’s clinical trial investigating GA-AMD [45], Tandospirone showed no benefit in relation to macular lesion progression compared to controls.

### 4.7. Recombinant Human Nerve Growth Factor

Beykin, in 2022 [47], also found no effect for topical Recombinant Human Nerve Growth Factor in glaucoma patients. However, the authors suggest that their study could be underpowered.

### 4.8. Prostaglandins

Isopropyl Unoprostone (Rescula), 2×/day, is a standard treatment for glaucoma patients. Tawada [44] used Rescula in retinitis pigmentosa patients, based on neuroprotection effect and the increase of retinal and choroidal circulation. At 6 months of follow-up, both BCVA and microperimetry were improved. The number of eyes was small (30), however, as was the case for the other neuroprotector that showed success, Citicoline (24 patients). For comparison, Tandospirone (unsuccessful) was investigated on 508 eyes.

### 4.9. Coenzyme Q10

Coenzyme Q10 0.1%, 2×/day, for 6 months, proved effective in Alzheimer’s patients, targeting retinal ganglion cells, and increasing retinal nerve fiber layer on OCT. Coenzyme Q10 has activity on mitochondrial dysfunction, oxidative stress and chronic neuro-inflammation.

### 4.10. Mecamylamine

Mecamylamine is a nonspecific nicotinic acetylcholine (nACh) receptor antagonist that was approved as an oral antihypertensive agent in the 1950s. It is known to pass through blood-brain barrier. Blocking nACh receptor could reduce abnormal vascular permeability in patients with DME. The effect was tested on animal settings, using topical concentrations varying 0.1 to 1%. Campochiaro’s study showed no local side effects on Mecamylamine topical administration (1% concentration, 2×/day) in patients with DME [48]. The clinical results were however mixed. From 21 patients, 8 showed improvement (in terms of BCVA and OCT-CRT); 9 had no change; while 4 patients showed mecamylamine-induced worsening. The deterioration was linked to drug-effect, taking place at 1 week after topical treatment initiation. The authors’ hypothesis for the results was the presence of mixed types of nACh receptors, some having opposite effects on vascular permeability.

### 4.11. Squalamine

Squalamine lactate, an angiostatic aminosterol interferes with the calmodulin mediated signaling for VEGF, platelet-derived growth factor, basic fibroblast growth factor (bFGF) and hepatocyte growth factor (HGF). Adding Squalamine to Ranibizumab (selective inhibitor of VEGF) may benefit from the inhibition of the other cytokines (bFGF, HGF) as well. Wroblewski et al. [50] treated 20 patients suffering from various forms of retinal vein occlusion. Topical Squalamine alone could not improve CME. Squalamine and Ranibizumab IVT treatment proved superior to Ranibizumab Pro Re Nata protocol alone (better vision, thinner retinas). However, the addition of Squalamine could not reduce the IVT injection burden.

### 4.12. Interferons

Interferons have antiproliferative, antiangiogenic and immunomodulatory properties. This makes Interferon a suitable candidate for the treatment of DME, and systemic use was proven effective in inflammation-related CME [90]. Retrobulbar injection in rabbit eyes led to important concentration of the drug in the choroid. This suggests a scleral route for entering the eye. Afarid [49] took the idea further and investigated the effect of topical IFNa2b in the treatment of DME. The results showed improved BCVA and non-statistically significant decrement of OCT-CRT.

### 4.13. Present Limitations and Future Directions

Some of the inconsistencies between the encouraging results obtained on animal models and the unsatisfactory outcome of certain subsequent clinical studies may reside on the lack of linear translation in between different mammals. Significant differences between species could influence both pharmacokinetic parameters and efficacy of topically applied drugs in ophthalmology and could affect the translation of promising data obtained in preclinical settings to clinical use. The eye size is variable between different species with a vitreous volume of 0.013–0.054 mL in rats, 1.5–1.8 mL in rabbits and 4 mL in humans, leading to differences in apparent volume of distribution and elimination of half-lives in the vitreous [91].

The study of Sadeghi et al. [92] showed that a dose scaling of 2–5 should be used in rabbits to achieve similar drug concentrations of macromolecules in the posterior eye segment, as compared to rats, due to different vitreal residence times. Furthermore, dose scaling in humans is still under debate, with no clear conclusions being reached yet.

The studies presented here took place over a short period of time and included a relatively small number of subjects, which does not cover the medical need, considering the incidence of retinal diseases. Many of the published results refer to the clinical efficiency or to the drug concentration in vitreous, as a result of the comparative testing of different pharmaceutical dosage forms, as well as two different APIs, administered by different routes (e.g., topical NSAID treatment, associated with intravitreal injection of a corticosteroid or monoclonal antibodies), a fact that does not confirm the effectiveness of these preparations or molecules.

On the other hand, the concentration of the APIs identified in the vitreous, at relatively short time intervals of application, is not relevant for formulating conclusions regarding the use of the respective molecules in chronic treatment, especially since their kinetics in the eye are not known for the long term. In addition, considering the high sensitivity of the anatomical structures at the level of the eye, long-term testing of the toxicity of the preparations in the eye is also required. Also, comparing the results obtained in different studies, using APIs from different generations, or surgical techniques, instruments, equipment and even different patients’ samples, is not relevant and may mislead in formulating some conclusions.

Despite the considerable scientific reasons, the existing evidence is insufficient, as convincing clinical results are needed to recommend a certain type of preparations and active principles for chronic clinical use in retinal diseases. Nevertheless, the prospects of bringing more and more sensitive imaging and spectroscopic tools able to locally monitor the API’s ocular biointernalization, its kinetics and even record changes upon the applied topical treatment in the pathophysiological profile at metabolites level could bring an extra boost in the in vivo assessment of the (pre)clinical efficacy of this route of administration on retinal diseases. Magnetic resonance imaging and spectroscopic techniques (MRI/MRS) are widely used in humans both for clinical diagnostic applications and in basic research areas [93]. Such an analytical hyphenation would offer a non-invasive approach, reducing the need for invasive procedures and minimizing the risk of complications. Additionally, the high spatial (anatomic) and chemical resolution of MRI/MRS allows a detailed analysis of tissue structure and metabolic activity and also presents a quantitative feature, providing an accurate assessment and comparison of data. Moreover, the translation of MRS to clinical practice tends to become more widely accepted once a set of minimum reporting standards were set in place [94].

The numerous biological and functional barriers of the eye rose too many challenges for topical translation of the drug molecules originally developed for systemic or parenteral administration, rendering the topical route of administration less appealing for ocular drug development. However, the relatively small number of successful pre-clinical (animal models) and clinical studies demonstrated that these barriers can in fact be efficiently circumvented, at least in case of selected representatives of drug classes. The development of a product intended for topical application at the level of the eye to target the retina represents a major challenge, considering the advantages of this non-invasive and economical way of administration. On the other hand, it is imperative to achieve an optimal combination between the system in which the API is incorporated or processed (nanosystems, inclusion complexes, crystalline polymorphs, salts, co-crystals), the dosage form and the physicochemical and pharmacokinetic characteristics of the API [3,95,96]. In the achieving of this objective, prolonging the retention time of the system on the ocular surface, ensuring a constant release of an adequate dose of API and using APIs with a strong pharmacodynamic activity represent key elements in establishing the preparation technology of the dosage form, considering that, after topical application, the level of concentrations in the retina will be in the nanomolar or picomolar range. An important aspect is also represented by the kinetics of APIs, as well as of the formulation excipients in the eye, as metabolic products can have an important impact on the anatomical structures of the eye.

This development could lead eventually to a gradual replacement in the current clinical practice of the more invasive modes of administration of APIs (IVT, sub-tenon, etc.) with the more convenient and patient-friendly topical route, except maybe those requiring immediate interventions.

## 5. Conclusions

Topical administration of drugs to the eye surface is easy to perform and does not induce significant discomfort, having very good acceptance rates from patients, compared to other more invasive routes of ocular drug administration.

Currently, the topical treatment of retinal degenerative diseases is limited by the difficulty to deliver effective drug concentrations to the posterior eye structures. However, in case of drug classes like NSAIDs, the presence of certain molecular and metabolic features for specific representatives makes the topical administration possible and feasible in several clinical contexts.

For other drug classes, several formulation strategies like the use of nanostructured drugs and vehicles, crystalline polymorphs or supramolecular complexes could enhance drug diffusion towards retinal structures and could be employed in the development of more effective ways of delivering drugs to the posterior eye, with the ultimate goal of improving their clinical efficacy.

## Figures and Tables

**Table 1:** Selection of APIs used for the treatment of retinal pathologies. Administration routes, main barriers in reaching target and main disadvantages.

Drug Class	Representatives	Routes of Administration	Main Barrier to Target	Main Disadvantages
Antioxidants	Lutein, Zeaxanthin (& derivatives), Vitamins C and E	Systemic—oral	Blood-retinal	Low bioavailability, low intestinal absorption
Anti-inflammatory: NSAIDs	Bromfenac, Nepafenac	Topical	Corneal	Low bioavailability
Anti-inflammatory: Corticosteroids	Triamcinolone	IVT	Internal limiting membrane	Surgical procedure (invasive)
	Metilprednisolone Dexamethasone	Oral or i.v. Subtenon injection i.v.	Blood-retinal Scleral Blood-retinal	Systemic adverse reactions, low bioavailability Invasive, low bioavailability, systemic adverse reactions Systemic adverse reactions, low bioavailability
Anti-VEGF	Bevacizumab, Aflibercept, Brolucizumab, etc.	IVT	Internal limiting membrane	Surgical procedure (invasive)

IVT—intravitreal; i.v.—intravenous.

**Table 2:** Topical treatment for degenerative retinal diseases in clinical trials, grouped in medicine classes and in chronological order.

Drug and Regimen	Proposed Mechanism	Pathology Studied	No. of Eyes Treated	Duration	Main Outcomes	Declared Success	Ref.
Bromfenac 2×/day, 12 months (plus Ranibizumab IVT, 1×/month—4 months, then as needed)	NSAID, Prostaglandin synthesis inhibitor	nAMD	20	12 months	BCVA similar; Need of intravitreal injection similar; OCT-CRT lower than in control group **	Partial	[17]
Bromfenac 2×/day, 6 months, (plus at least 1 dozen Ranibizumab IVT), versus sham (Ranibizumab, only)	NSAID Prostaglandin synthesis inhibitor	nAMD	15	6 months	Need of intravitreal injection 2.2 versus 3.2; BCVA similar; OCT-CRT similar	Partial	[18]
Ketorolac 0.5%, versus Acetazolamide 250 mg/day, and control	NSAID Prostaglandin synthesis inhibitor	CME after cataract surgery	27	1 month	BCVA, OCT-CRT better in Ketorolac and Acetazolamide group, versus control **; No difference Ketorolac versus Acetazolamide	Yes	[19]
Ketorolac 0.45%, 3×/day, 6 months plus Ranibizumab 0.5 mg IVT (1×/month—3 month, then on demand), versus Ranibizumab alone	NSAID Prostaglandin synthesis inhibitor	nAMD	28	6 months	BCVA similar in the 2 groups; Need for IVT, similar; OCT-CRT—Ketorolac combination with greater reduction **	Yes	[20]
Bromfenac 0.09% 2 × 1 (plus Bevacizumab 1.25 mg, IVT); control Bevacizumab 1.25 mg, IVT	NSAID Prostaglandin synthesis inhibitor	nAMD	26	6 months	BCVA, OCT-CRT better than control (Bevacizumab only) **	Yes	[21]
Nepafenac, 0.1%, 3×/day	NSAID Prostaglandin synthesis inhibitor	DME (noncentral)	61	12 months	BCVA, OCT-CRT—no difference from baseline and versus placebo	No	[22]
Ketorolac 0.4% or Nepafenac 0.1% versus placebo	NSAID Prostaglandin synthesis inhibitor	CME after uncomplicated cataract surgery	84	3 months	BCVA, OCT-CRT not different between groups; 2.1%, 2.4% and 2.9% of CME also at postoperative 4 weeks in the placebo, ketorolac and nepafenac groups, respectively	no	[23] *
Ketorolac 0.5% 4 × 1 (1 week)	NSAID Prostaglandin synthesis inhibitor	Macular edema after Nd:YAG laser capsulotomy	44	6 months	OCT—CRT lower versus control ** (Fluorometholone 0.1%)	Yes	[24]
Diclofenac 0.1%, 4×/day, preop and 6 weeks after phacoemulsification	NSAID, Prostaglandin synthesis inhibitor	Diabetic cataract—profilaxy of CME after cataract surgery	54	3 months	BCVA similar in treated versus not treated; OCT-CRT lower in treated **	Partial	[25]
Nepafenac 0.1%, 2 × 1, versus Subtenon Triamcinolone.	NSAID Prostaglandin synthesis inhibitor; Decrease VEGF mRNA	CME after cataract surgery	24	6 months	BCVA increase **; OCT -CRT decreased **; Effect better then control	Yes	[26]
Nepafenac 0.3%, 1×/day, 5 weeks, versus placebo	NSAID Prostaglandin synthesis inhibitor	CME after uncomplicated cataract surgery	503	1.5 months	CME significantly reduced in patients with preoperative risk factors **	Yes	[27] *
Nepafenac 0.1%, 3x/day, or Bromfenac 0.09%, 2×/day, Control Dexamethasone	NSAID Prostaglandin synthesis inhibitor	CME after uncomplicated cataract surgery	96	1 month	No CME in treated groups (both Nepafenac and Bromfenac) BCVA, OCT-CRT similar in treated and controls	Partial	[28]
Nepafenac 0.3%, bilateral surgery (one eye treaded, the other eye placebo); 30 days between surgeries of the 2 eyes	NSAID Prostaglandin synthesis inhibitor	CME after uncomplicated cataract surgery	112	3 months	OCT-CRT improved in treated, at 5 weeks **; No CME in treated, at 5 weeks (versus 3.57%) BCVA similar	Yes	[29]
Nepafenac 0.1%, 3 × 1/day, 4 weeks, versus 1 dose Ranibizumab 0.5 mg, IVT at surgery	NSAID Prostaglandin synthesis inhibitor	Diabetic cataract—prophylaxis of CME after cataract surgery	38	3 months	OCT-CRT—preserved, same effect as Ranibizumab	Yes	[30]
Prednisolone acetonide high dose—every hour, versus low dose—4×/day (plus ketorolac)	Corticosteroid-Phospholipase A2 inhibitor.	CME after cataract surgery	22	4 months	BCVA similar in high and low dose; OCT-CRT—similar in high and low dose	No	[31] *
Dexamethasone-Cyclodextrin Microparticles 1.5%, 3 × 1/day, or 6 × 1/day, 4 weeks	Corticosteroid-Phospholipase A2 inhibitor	DME	19	2 months	BCVA, OCT-CRT improvement at week 4 **; BCVA returned to baseline at week 8, OCT-CRT remained decreased **	Yes	[32] *
Dexamethasone-cyclodextrin nanoparticles 1.5%, 1×/day, or 2 × 1/day, or 3 × 1/day (1 month)	Corticosteroid-Phospholipase A2 inhibitor	DME	12	3 months	BCVA improvement; OCT-CRT decreased. ** Effect comparable to Triamcinolone (subtenon injection)	Yes	[33]
Dorzolamide, 3×/day	Carbonic anhydrase inhibition	CME in retinitis pigmentosa	16	6 months	OCT-CRT decrease in 81% **; Visual field improvement **	Yes	[34]
Dorzolamide 2×/day; Ketorolac 0.5% 4×/day	Carbonic anhydrase inhibition; NSAID	CME in retinitis pigmentosa	13 Dorzolamide 15 Ketorolac	12 months	BCVA increase (both’s treatment; unclear result at 12 months for Dorzolamide); OCT-CRT unchanged	Partial	[35]
Dorzolamide (Trusopt), 2×/day, 7 days	Carbonic anhydrase inhibition	Normals; DR	41 (20 with diabetic retinopathy)	7 days	Dynamic vessel analyzer -Dilated vessels in normal subjects ** -No effect in diabetic	Yes	[36]
Dorzolamide (Trusopt), 4×/day, 3 months	Carbonic anhydrase inhibition; may improve subretinal fluid absorption through the RPE	Chronic Central Serous Chorioretinopathy	18	3 months	BCVA similar to controls; OCT-CRT decreased **; Subretinal fluid resolution (77.8% versus 40% in controls) **	Yes	[37]
Dorzolamide-Timolol, 2×/day, versus placebo (in parallel to antiVEGF IVT regimen, as needed)	Carbonic anhydrase inhibition; beta-blocker	nAMD	27	3 months	OCT-CRT lower in treated **; BCVA similar	Yes	[38] *
Coenzyme Q10 0.1%, 2×/day, 6 months	Reduction of mitochondrial disfunction, oxidative stress, chronic neuro-inflammation	Alzheimer’s disease with visual function deteriorations	30	6 months	OCT RNFL increase **	Yes	[16]
Integrin inhibitor (OTT166, 2.5/5%), 2×/day, 28 days	Decrease angiogenesis, exudation, inflammation, and fibrosis (RGD binding integrins including αvβ3, αvβ6, and αvβ8)	Diabetic retinopathy; DME	44	2 months	OCT-CRT reduction in 37% (responders) **	Partial	[39] *
Pazopanib, 5 mg/mL, 3 × 1/day, or 1 × 1/day, or 2 mg/mL 3 × 1/day, 28 days1	Multitarget tyrosine kinase inhibitor—all VEGF receptor subtypes, and platelet-derived growth factor	nAMD	68	1 months	BCVA improvement in 5 mg/mL 3 × 1/day group **; OCT-CRT improvement in CFH T allele genotype subset of AMD **	Yes	[40] *
Pazopanib 10 mg/mL, 4 × 2/day, or 4 × 1/day, 2 weeks (study 1), 4 × 1/day, 12 weeks (study 2), versus placebo	Multitarget tyrosine kinase inhibitor—all VEGF receptor subtypes, and platelet-derived growth factor	nAMD	34 (study 1) 19 (study 2)	3 months	Well tolerated; BCVA, OCT-CRT not changed; Study 2–9 patients with rescue therapy (IVT)	No	[41]
Regorafenib, 25 μL, 30 mg/mL, 3×/day, 3 months	Multikinase inhibitor—VEGF receptor 2/3, and platelet-derived growth factor receptor β	nAMD	51	3 months	BCVA decreased **; rescue IVT in 20 patients	No	[42] *
Acrizanib (LHA510) 2%, 2 × 1/day—8 weeks, then 3 × 1/day—4 weeks	Tyrosine kinase-VEGF receptor inhibitor	nAMD	33	3 months	OCT- macular fluid accumulation; Ranibizumab IVT (rescue) needed same as in placebo group	No	[43]
Isopropyl Unoprostone (Rescula), 2×/day	Increased retinal and choroidal circulation; Neuroprotection	Retinitis pigmentosa	30	6 months	Microperimetry improved; ** BCVA improved **	Yes	[44]
Tandospirone 1%, or 1.75%, 2×/day, versus vehicle	Neuroprotection (agonist on 5-HT1A receptor)	GA-AMD	508	30 months	Lesion growths similar—1.73, 1.76, and 1.71 mm^2^ for 1.0%, 1.75% and vehicle	No	[45] *
Citicoline, 3×/day, versus beta-blockers	Neuroenhancement (stabilizes cell membranes by increasing phosphatidylcholine and sphingomyelin synthesis).	Glaucoma	24	4 months	VEP, ERG—increased amplitudes, shortened latency **; Values returned to baseline 2 months after Citicoline washout	Yes	[46]
Recombinant Human Nerve Growth Factor (rhNGF), 180 μg/mL, 3×/day—8 weeks, versus vehicle control	Neuroenhancement, retinal ganglion cell survival.	Glaucoma	40	8 months	No adverse reactions; OCT-RNFL and visual field, similar to control	No	[47] *
Mecamylamine 1%, 2×/day	Nonspecific nicotine receptor antagonist	DME	21	4 months	8 eyes—improvement (BCVA, OCT-CRT) 4 eyes—worse (BCVA, OCT-CRT)	Mixed	[48] *
Interferon α2b 1 million U/mL, 4×/day, 4 weeks	Antiproliferative antiangiogenic and immunomodulatory properties.	DME	25	2 months	BCVA increased; OCT-CRT decreased (statistically non-significant, versus placebo—artificial tears)	Partial (objective criteria not met)	[49]
Squalamine 2×/day, 10 weeks, plus Ranibizumab 0.5 mg IVT as needed. After week 10, 2 groups were formed (continued Squalamine 2×/day, or not)	Angiostatic aminosterol; Inhibition of VEGF, PDGF, basic fibroblast growth factor (bFGF), and hepatocyte growth factor (HGF)—impact on endothelial cell, angiogenesis	CME in Retinal vein occlusion	20	6 months	Combination therapy improved BCVA outcome; Squalamine alone did not improved CME	Partial	[50]

* registered at ClinicalTrials.gov; ** *p* value < 0.05. NSAID—nonsteroid anti-inflammatory drugs, CME—cystoid macular edema, DR—diabetic retinopathy, DME—diabetic macular edema, nAMD—neovascular age related macular degeneration, GA-AMD—geographic atrophy in age related macular degeneration, BCVA—best corrected visual acuity, OCT—ocular coherence tomography, CRT—central retinal thickness, VEP—visual evoked potential, ERG—electroretinogram, IVT—intravitreal injection, CFH—complement factor H.

## Data Availability

Not applicable.
